# Supplementary material for: Heat adaptation of phage T7 under an extended genetic code
Source: Virus Evol. 2021 Dec 1;7(2):veab100. doi: 10.1093/ve/veab100 (PMC8923235; doi:10.1093/ve/veab100)
Supplement: veab100_Supp [file veab100_supp.zip › Sequencing_Analysis.zip!/Sequencing_Analysis/A1/evidence/JC_SIDE_2_63.html]

BRESEQ :: Evidence


| New junction evidence | | | | | | | | | | | |
| --- | --- | --- | --- | --- | --- | --- | --- | --- | --- | --- | --- |
|  | | seq id | position | reads (cov) | reads (cov) | score | skew | freq | annotation | gene | product |
| \* | ? | NC\_001604.1 | = 16604 | 2883 (0.930) | 10 (0.000) | 9/590 | 41.6 | 0.35% | intergenic (–/–) | *–/–* | –/– |
|  | ? | NC\_001604.1 | 17028 = | 2874 (0.950) |  |  |  |  | intergenic (–/–) | *–/–* | –/– |
| Rejected: Coverage evenness skew score above cutoff. | | | | | | | | | | | |
| Rejected: Frequency below/above cutoff threshold. | | | | | | | | | | | |

Only 100 of 3047 total aligned reads displayed.  

`TGACATTCTTGCGGTTGTGGTTGACGAAGATGTGCTTATTTTCACATGGGACGAAGTAAAAGGTAAGACATCCATGTGTGTCGGCAAGAGAAACAAAGGCATAAAACTATAGGAGAAATTATTATGGCTATGACAAAGAAATTTAAAGTGTCCTTCGACGTTACCGCAAAGATGTCGTCTGACGTTCAGGCAATCTTAGAGAAAGATATGCTGCATCTATGTAAGCAGGTCGGCTCAGGTGCGATTGTCCCCAATGGTAAACAGAAGGAAATGATTGTCCAGTTCCTGACACACGGTATGGAAGGATTGATGACATTCGTAGTACGTACATCATTTCGTGAGGCCATTAAGGACATGCACGAAGAGTATGCAGATAAGGACTCTTTCAAACAATCTCCTGCAACAGTACGGGAGGTGTTCTGATGTCTGACTACCTGAAAGTGCTGCAAGCAATCAAAAGTTGCCCTAAGACTTTCCAGTCCAACTATGTACGGAACAATGCGAGCCTCGTAGCGGAGGCCGCTTCCCGTGGTCACATCTCGTGCCTGACTACTAGTGGACGTAACGGTGGCGCTTGGGAAATCACTGCTTCCGGTACTCG  >  NC_001604.1/16728‑17328`  

                                                                                                                                                                                                                                                                                                            `|`                                                                                                                                                                                                                                                                                                              
`tGATATTCTTGCGGTTGTGGTTGACGAAGATGTGCTTATTTTCACATGGGACGAAGTAAAAGGTAATACATCCATGTGTGTCGACAAGAGAAACAAAGGCATAAAACTATAGGAGAAATTATTATGGCTATGACAAAGAAATTTAAAGTGTCCTTCGACGTGACCGCAAAGATGTCGTCTGACGTTCAGGCAATCTTAGAGAAAGATATGCTGCATCTATGTAAGCAGGTCGGCTCAGGTGCGATTGTCCCCAATGGTAAACAGAAGGAAATGATTGTCCAGTTCCTGACACACGGTATgg                                                                                                                                                                                                                                                                                                              <  1:437220/301‑1 (MQ=255)`  
   `ctttCATGCGGTTGGGTTTGACGAATATGTGCTTATTTTCACATGGGACGAAGTAAAAGGTAAGACATCCATGTGTGTTGGCAAGGGAAACAAAGGCATAAAACTATAGGAGAAATTATTATGGCTATGACAAAGAAATTGAAAGTGTCCTTCGACGTTACCGCAAAGATGTCGTCTGACGTTCAGGCAATCTTAGAGAAAGATATGCTGCATCTATGTAAGCAGGTCGGCTTAGGTGCGATTGTCCCCAATGGTAAACAGAAGGAAATGATTGTCCAGTTCCTGACACACGGTATGGAAg                                                                                                                                                                                                                                                                                                           <  1:192407/299‑1 (MQ=255)`  
   `cATTCTTGCGGTTGTGGTTGACGAAGATGTGCTTATTTTCACATGGGACGAAGTAAAAGGTAAGACATCCATGTGTGTCGGCAAGAGAAACAAAGGCATAAAACTATAGGAGAAATTATTATGGCTATGACAAAGAAATTTAAAGTGTCCTTCGACGTTACCGCAAAGATGTCGTCTGACGTTCAGGCAATCTTAGAGAAAGATATGCTGCATCTATGTAAGCAGGTCGGCTCAGGTGCGATTGTCCCCAATGGTAAACAGAAGGAAATGATTGTCCAGTTCCTGACACACGGTATGGAAg                                                                                                                                                                                                                                                                                                           <  1:223598/301‑1 (MQ=255)`  
       `cTTGTGGTTGTGGTTGACGAAGATGTGCTTATTTTCACATGGGACGAAGTAAAAGGTAAGACATCCATGTTTGTCGGCATGAGCAACATAGACATAAAATTATAGGAGAAATTATTATGGCTGTGACATAGTAATTTATTTTGTCGTTCTCCGTTGCCGCAACACTGTCGTCTGACGTTCAGGCAATCTTAGTGAAAGTTATGCTGCATCTGTTTAAGCTGGTTGCTTCACGTGAAACTGTCGTAATTGGTTAACTAAGTGTACTGTTTTTGCAGCTGCTGACACCTGGTAAGGAAGGAtt                                                                                                                                                                                                                                                                                                       >  1:466692/1‑301 (MQ=255)`  
        `ttGCGGTTGTGGTTGACGAAGATGTGCTTATTTTCACATGGGACGAAGTAAAAGGTAAGACATCCATGTGTGTCGGCAAGAGAAACAAAGGCATAAAACTATATGAGAAATTATTATGGCTATGACAAAGAAATTTAAAGTGTCCTTCGACGTTACCGCAAAGATGTCGTCTGACGTTCAGGCAATCTTAGAGAAAGATATGCTGCATCTATGTAAGCAGGTCGGCTCAGGTGCGATTGTCCCCAATGGTAAACAGAAGGAAATGATTGTCCAGTTCCTGACAAACGGTATGGAAGGATtg                                                                                                                                                                                                                                                                                                      >  1:377523/1‑301 (MQ=255)`  
             `gTTGTGGTTGACGAAGATGGTCTTATTTGCACATTGGACGAAGTAAAAGGTAAGACATCCATGTGTGTCGGCAAGAGAAACAAAGGCATAAAACTATAGGAGAAATTATTATGGCTATGACAAAGAAATTTAAAGTGTCCTTCGACGTTACCGCAAAGATGTCGTCTGACGTTCAGGCAATCTTAGAGAAAGATATGCTGCATCTATGTAAGCAGGTCGGCTCAGGTGCGATTGTCCCCAATGGTAAACAGAAGGAAATGATTGTCCAGTTCCTGACACACGGTATGGAAGGATTGATGAc                                                                                                                                                                                                                                                                                                 <  1:76329/301‑1 (MQ=255)`  
                   `gTTGACGAAGATGTGCTTATTTTCACATGGGACGAAGTAAAAGGTAAGACATCCATGTGTGTCGGCAAGAGAAACAAAGGCATAAAACTATAGGAGAAATTATTATGGCTATGACAAAGAAATTTAAAGTGTCCTTCGACGTTACCGCAAAGATGTCGTCTGACGTTCAGGCAATCTTAGAGAAAGATATGCTGCATCTATGTAAGCAGGTCGGCTCAGGTGCGATTGTCCCCAATGGTAAACAGAAGGAAATGATTGTCCAGTTCCTGACACACGGTATGGAAGGATTGATGACATTCgt                                                                                                                                                                                                                                                                                           >  1:21876/1‑301 (MQ=255)`  
                     `tGACGACGGTGTTATTATTTTCACAGGGGACGAAGTAAAAGGTAAGACATCCATGTGTGTCGGCAAGAGAAACAAAGGCATAAAACTAAAGGAGACATTATGATGGCTATGACAAAGAAATTTAAAGTGTCCTTCGACGTTACCGCAAAGATGTCGTCTGACGTTCAGGCAATCTTAGAGAAAGATATGCTGCATCTATGTAAGCAGGTCGGCTCAGGTGCGATTGTCCCCAATGGTAAACAGAAGGAAATGATTGTCCAGTTCCTGACACACGGTATGGAAGGATTGATGACATTCgtag                                                                                                                                                                                                                                                                                         <  1:428816/301‑1 (MQ=255)`  
                     `tGACGAAGATGGGCTTATTTTCACATGGGACGAAGTAAAAGGTAAGACAGCCATGTGTGTCGGCAAGAGAAACAAAGGCATAAAACGATAGGAGAAATTATTATGGCTATGACAAAGAAATTTAAAGTGTCCTTCGACGTTACCGCAAAGATGTCGTCTGACGTTCAGGCAATCTTAGAGAAAGATATGCTGCATCTATGTAAGCAGGTCGGCTCAGGTGCGATTGTCCCCAATGGTAAACAGAAGGAAATGATTGTCCAGTTCCTGACACACGGTATGGAAGGATTGATGACATTCgtag                                                                                                                                                                                                                                                                                         <  1:198662/301‑1 (MQ=255)`  
                       `aagagacagtgCGTATTTTCACATGGGACGAAATAAAAGGTAAGACATCCATGTGTGTCGGCAAGAGAAACAAAGGCATAAAACTATAGGAGAAATTATTATGGCTATGACAAAGAAATTTAAAGTGTCCTTAGACGTTAACGCAAAGATGTCGTCTGACGTTCAGGCAATCGTAGAGAAAGATATGCTGCATCTATGTAAGCAGGTCGGATCAGGTGCGATTGTCCCCAATGGTAAACAGAAGGAAATGATTGTCCAGTTCCTGACACACGGTATGGAAGGATTGATGACATTCgtagta                                                                                                                                                                                                                                                                                       <  1:38808/293‑1 (MQ=255)`  
                       `aCGAAGATGTTCTTATTTGCACATGGGACGAAGTAAAAGGTAAGACATCCATGTGTGTCGGCAAGAGAAACAAAGGCATAAAACTATAGGAGAAATTATTATGGCTATGACAAAGAAATTTAAAGTGTCCTTCGACGTTACCGCAAAGATGTCGTCTGACGTTCAGGCAATCTTAGAGAAAGATATGCTGCATCTATGTAAGCAGTTCGGCTCAGGTGCGATTGTCCCCAATGGTAAACAGAAGGAAATGATTGTCCAGTTCCTGACACACGGTATGGAAGGATTGATGACATTCgtagta                                                                                                                                                                                                                                                                                       <  1:431918/301‑1 (MQ=255)`  
                                   `atATTTGCACAGGGGACGAAGTAAAAGGTAAGAAATCCATGTGTGTCGGCAAGAGAAACAAAGGCATAAAACTATAGGAGAAATTATTATGGCTATGAAAAAGAAATTTAAAGTGTCCTTCGACGTTACCGCAAAGATGTCGTCTGACGTTCAGGCAATCTTAGAGAAAGATATGCTGAATCTATGTAAGCAGGTCGGCTCAGGTGCGATTGTCCCAAATGGTAAACAGAAGGAAATGATTGTCCAGTTCCTGACACACGGTATGGAAGGATTGATGACATTCGTAGTACGTACATCAttt                                                                                                                                                                                                                                                                           <  1:157246/300‑1 (MQ=255)`  
                                       `aagagaaagggACGAAGTAAAAGGTAAGACATACATGTGTGTAGGCAAGAGAAACAAATGCATAAAACTATATGAGAAATTATTATGGCTATGACAAAGAAATTTAAAGTGTCCTTCGACGTTACCGCAAAGATGTCGTCTGACGTTCAGGCAATCTTATAGAAAGATATGCTGCATCTATGTAAGCAGGTCGGCTCAGGTGCTATTGTCCCCAATGGTAAACAGAAGGAAATGATTGTCCAGTTCCTGACACACGGTATGGAAGGATTGATGACATTCGTAGTACGTACATCATTTCGTg                                                                                                                                                                                                                                                                       <  1:124540/293‑1 (MQ=255)`  
                                         `tCACATGGGACGAAGTAAAAGGTAAGACATCCATGTGTTTCGGCAAGAGAAACAAAGGCATAAAACTATAGGAGAAATTATTATGGCTATGACAAAGCAATTTAAAGTGTCCTTCGACGTTACCGCAAAGATGTCGTCTGACGTTCAACCAATCTTAGAGAAAGCTATGCTGCATCTATGTACGCAGGTCGCCTCACGTGCGATTCTCCCCCATGGTAAACAGAAGGAAATGCATGTCCAGTTCCTGACCCCCGGTATGGAAGGATTGATGCCCTTCGTAGTCCGTACATCCTTTcttcat                                                                                                                                                                                                                                                                     >  1:55381/1‑296 (MQ=255)`  
                                            `aaTGGGACGAAGTAAAAGGTAAGACATCCATGGGTGTCGGAAAGAGAAACAAAGGCATAAAACTATAGGAGAAATTATTATGGCTATGACAAAGAAATTTAAAGTGTCCTTCGACGTTACCGCAAAGAGGTCGTCTGACGTTCAGGCAATCTTAGAGAAAGATATGCTGCATCTATGTAAGCAGGTCGGCTCAGGTGCGATTGTCCCCAATGGTAAACAGAAGAAAATGATTGTCCAGTTCCTGACACACGGTATGGAAGGATTGATGACATTCGTAGTACGTACATCATTTCGTGAGGcc                                                                                                                                                                                                                                                                  <  1:231515/300‑1 (MQ=255)`  
                                                    `caggTAAAAAGTAAGAAATTCATGTGTGTCGGCAAGAGAAACAAAGGCATAAAACTATAGGAGAAATTATTATGGCTATGACAAAGAAATTTAAAGGGTCCTTCGACGTTACCGCAAAGATGTCGTCTGACGTTCAGGGAATCTTAGAGAAAGATATGCTGCATCTATGTAAGCAGGTCGGCTCAGGTGCGATTGTCCCCAATGGTAAACAGAAGGAAATGATTGTCCAGTTCCTGACACACGGTATGGAAGGATTGATGACATTCGTAGTACGTACATCATTTCGTGAGGCCATTAAGGa                                                                                                                                                                                                                                                          <  1:350908/298‑1 (MQ=255)`  
                                                    `aaggaaaaagtaaagaagtacATGTGTGACGGCAAGAGAAACAAAGGCATAAAACTATAGGAGAAATTATTATGGCTATGACAAAGAAATTTAAAGTGTCCTTTGACGTTACCGCAAAGATGTCGTCTGACGTTCAGGCAATCTTAGAGAAAGATATGCTGCATCTATGTAAGCAGGTCGGCTAAGGTGCGATTGTCCCCAATGGTAAACAGAAGGAAATGATTGTCAAGGTCCTGACATACGGTATGGAAGGATTGATGACATTCGTAGTACGTACATCATTGCGTGAGGCCATTAAGGa                                                                                                                                                                                                                                                          <  1:399082/281‑1 (MQ=255)`  
                                                     `aaGTAAAAGGTAAGACATCCATGTGTGTCGGCAAGAGAAACAAAGGCATAAAACTATAGGAGAAATTATTATGTCTATGACAAAGAAATTTAAAGTGTCCTTCGACGTTACCGCAAAGATGTCGTCTTACGTTCAGGCAATCTTAGAGAAAGATATGCTGCATCTATGTAAGCAGGTCGGCTCAGGTGCGATTGTCCCCAATGGTAAACAGAAGGAAATGATTGTCCAGTTCCTGACACACGGTATGGAAGGCTTGATGACATTCGTAGTACGTAAATCATTTCGTGAGGCCCTTAAGGAc                                                                                                                                                                                                                                                         >  1:419401/1‑301 (MQ=255)`  
                                                     `aaGTAAAAGGTAAGACATCCATGTGTGTCGGCAAGAGAAACAAAGGCATAAAACTATAGGAGAAATTATTATGGCTATGACAAAGAAATTTCAAGTGTCCTTCGACGTTACCGCAAAGATGTCGTCTGACGTTCAGGCAATCTTAGAGAAAGATATGCTGCATCTATGTAAGCAGGTCGGCTCAGGTGCGATTGTCCCCAATGGTAAACAGAAGGAAATGATTGTCCAGTTCCTGACACACGGTATGGAAGGATTGATGACATTCGTAGTACGTACATCATTTCGTGAGGCCATTAAGGAc                                                                                                                                                                                                                                                         >  1:403652/1‑301 (MQ=255)`  
                                                      `aGTAAAAGGTAAGACATCCATGTGTGTCGGCAAGAGAAACAAAGGCATAAAACTATAGGAGAAATTATTATGGCTATGACAAAGAAATTTAAAGTGTCCTTCGACGTTACCGCAACGATGTCGTCTTACGTTCAGGCAATCTTAGAGAAAGATATGCTGCATCTATGTAAGCAGGTCGGCTCAGGTGCGATTGTCCCCAATGGTAAACAGAAGGAAATGATTGTCCAGTTCCTGACACACGGTATGGAAGGATTGATGACATTCGTAGTACGTACATCATTTCGTGAGGCCATTAAGGACa                                                                                                                                                                                                                                                        <  1:262150/301‑1 (MQ=255)`  
                                                             `ggTAAGACATCCATGTGTGTCGGCAAGAGAAACAAAGGCATAAAACTATAGGAGAAATTATTATGGCTATGACAAAGAAATTTAAAGTGTCCTTCGACGTTACCGCAAAGATGTCGTCTGACGTTCAGGCAATCTTAGAGAAAGATATGCTGCATCTATGTAAGCAGGTCGGCTCAGGTGCGATTGTCACCAATGGTAAACAGAAGGAAATGATTGTCCAGTTCCTGACACACGGTATGGAAGGATTGATGACATTCGTAGTACGTACATCATTTAGTGAGGCCATTAAGGACATACACGa                                                                                                                                                                                                                                                 >  1:291397/1‑301 (MQ=255)`  
                                                              `gTAAGACATCCATGTGTGTCGGCAAGAGAAACAAAGGCATAAAACTATAGGAGAAATTATTATGGCTATGACAAAGAAATTTAAAGTGTCCTTCGACGTTACCGCAAAGATGTCGTCTGACGTTCAGGCAATCTTAGAGAAAGATCTGCTGCAGCTATGTAAGCAGGTCGGCTCAGGTGCGATTGTCCCCAATGGTAAACAGAAGGAAATGATTGTCCAGGTCCTGACCCACGGTATGGAAGGATTGATGACATTCGTAGTACGTACATCATTTAGTGAGGCCATTACGGACATGCACGaa                                                                                                                                                                                                                                                >  1:510726/1‑301 (MQ=255)`  
                                                                  `gACATCCATGTGTGTCGGCAAGAGAAACAAAGGCATAAAACTATAGGAGAAATTATTATGGCTATGACAAAGAAATTTAAAGTGTCCTTCGACGTTACCGCAAAGATGTCGTCTGACGTTCAGGCAATCTTAGAGAAAGATATGCTGCATCTATGTAAGCAGGTCGGCTCAGGTGCGATTGTCCCCAATGGTAAACAGAAGGAAATGATTGTCCAGTTCCTGACACACGGTATGGAAGGATTGATGACATTCGTAGTACGTACATCATTTCGTGAGGCCATTAAGGACATGCACGAAGAGt                                                                                                                                                                                                                                            >  1:303189/1‑301 (MQ=255)`  
                                                                     `aTCCATGTGTGTCGGCAAGAGAAACAAAGGCATAAAACTATAGGAGAAATTATTATGGCTATGACAAAGAAATTTAAAGTGTCCTTCGACGTTACCGCAAAGATGTCGTCTGACGTTCAGGCAATCTTAGAGACAGATATGCTGCCTCTATGTAAGCCGGTCGGCTCAGGATCGCTTGTCCCCAATGGTAAACAGAAGGAACTGATTGTCCAGTTCCTGACCCACGCTATGGAAGGATTGATGACCTTCGTAGTACGTACATCCTTTCGTGAGGACATTAAGGACATGCACGAAGAGTAta                                                                                                                                                                                                                                         >  1:502235/1‑300 (MQ=255)`  
                                                                     `aTCCATGTGTGTCGGCAAGAGAAACAAAGGCATAAAACTATAGGAGAAATTATTATGGCTATGACAAAGAAATTTAAAGTGTCCTTCGACGTTACCGCAAAGATGTCGTCTGACGTTCAGGCAATCTTAGAGAAAGATATGCTGCGTCTATGTAAGCAGGTCGGCTCAGGTGCGATTGTCCCCAATGGTAAACAGAAGGAAATGATTGTCCAGTTCCTGACACACGGTATGGAAGGATTGATGACATTCGTAGTACGTACATCATTTCGTGAGGCCATTAAGGACATGCACGAAGAGTATg                                                                                                                                                                                                                                         >  1:213108/1‑301 (MQ=255)`  
                                                                     `aTCCATGTGTGTCGGCAAGAGAAACAAAGGCATAAAACTATAGGAGAAATTATTATGGCTATGACAAAGAAATTTAAAGTGTCCTTCGACGTTACCGCAAAGATGTCGTCTGACGTTCAGGCAATCTTAGAGAAAGATATGCTGCATCTATGTAAGCAGGTCGGCTCAGGTGCGATTGTCCCCAATGGTAAACAGAATGAAATGATTGTCCAGTTCCTGACACACGGTATGGAAGGATTGATGACATTCGTAGTACGTACATCATTTCGTGAGGCCATTAAGGACATGCACGAAGAGTATg                                                                                                                                                                                                                                         >  1:137097/1‑301 (MQ=255)`  
                                                                     `aTCCATGTGTGTCGGCAAGAGAAACAAAGGCATAAAACTATAGGAGAAATTATTATGGATATGACAAAGAAATTTAAAGTGTCCTTCGACGTTACCGCAAAGATGTCGTCTGACGTTCAGGCAATCTTAGAGAAAGATATGCTGCATCTATGTAAGCAGGTCGGCTCAGGTGCGATTGTCCCCAATGGTAAACAGAAGGAAATGATTGTCCAGTTCCTGACACACGGTATGGAAGGATTGATGACATTCGTAGTACGTACATCATTTCGTGAGGCCATTAAgctgtctcttatacacatct                                                                                                                                                                                                                                         >  1:470173/1‑282 (MQ=255)`  
                                                                      `accATGTGGTTCGGCAAAAGAAAAAAAGGTATAATACTATAGGAGAAATTATTATGGCTATGACAAAGAAATTTAAAGTGTCCTTCGACGTTACCGCAAAGATGTCGTCTGACGTTCAGGCAATCTTAGAGAAAGATATGCTGCATCTATGTAAGCAGGTCGGTTCAGGTGCGATTGTCCCCAATGGTAAACAGAAGGAAATGATTGTCCAGTTCCTGACACACGGTATGGAAGGATTGATGACATTCGTAGTACGTACATCATTTCGTGAGGCCATTAAGGACATGCACGAAGAGTATGc                                                                                                                                                                                                                                        <  1:227905/300‑1 (MQ=255)`  
                                                                          `tgtgtgTCGGCAAGAGAAACAAAGGCATAAAACTATAGGAGAAATTATTATGGCTATGACAAAGAAATTTAAAGTGTCCTTCGACGTTACCGCAAAGATGTCGTCTGACGTTCAGGCAATCTTAGAGAAAGATATGCTGCATCTATGTAAGCAGGTCGGCTCAGGTGCGATTGTCCCCAATGGTAAACAGAAGGAAATGATTGTCCAGTTCCTGACACACGGTATGGAAGGATTGATAACATTCGTAGTACGTACATCATTTCGTGAGGCCATTAAGGActgtctcttataccaatctccg                                                                                                                                                                                                                                    >  1:321264/1‑280 (MQ=255)`  
                                                                           `ttgtgtCGGCAAGAGAAACAAAGGCATAAAACTATAGGAGAAATTATTATGGCTATGACAAAGAAATTTAAAGTGTCCTTCGACGTTACCGCAAAGATGTCGTCTGACGTTCAGGCAATCTTAGAGAAAGATATGCTGCATCTATGTAAGCAGGTCGGCTCAGGTGCGATTGTCCCCAATGGTAAACAGAAGGAAATGATTGTCCAGTTCCTGACACACGGTATGGAAGGATTGATGACATTCGTAGTACGTACATCATTTCGTGAGGCCATTAAGGACATGCACGAAGAGTATGCAGATa                                                                                                                                                                                                                                   <  1:44971/300‑1 (MQ=255)`  
                                                                             `gtgtCGGAAAGAGAAACAAAGGCATAAAACTATAGGAGAAAGTATTATGGCTATGACAAAGAAATTTAAAGTGTCCTTCGACGTTACCGCAAAGATGTCGTCTGACGTTCAGGCAATCTTAGAGAAAGATATGCTGCATCTATGTAAGCAGGTCGGCTCAGGTGCGATTGTCCCCAATGGTAAACAGAAGGAAATGATTGTCCAGTTCCTGACACACGGTATGGAAGGATTGATGACATTCGTAGTACGTACATCATTTCGTGAGGCCATTAAGGACATGCACGAAGAGTATGCAGATAAg                                                                                                                                                                                                                                 <  1:356378/301‑1 (MQ=255)`  
                                                                                `agggCAAGATAAACAAAGGCATAAAACTATAGTAGAAATTATTCTGTCTATGACAAAGAAATTAAAAGTGTCCTTCGCCGTTACCGCAAAGATATTGTCTGACGTTCAGGCAATCTTAGAGAAAGATCTCCTGCATCTATGTAAGCAGGGCGGCTCAGGTGCGATTGTCCCTAATGGTAAACATAAGGAAATGATTGTCCAGTTCCTGACACACGGTATGGAAGGATTGATGACATTCGTAGTACGTACATCATTTCGTGAGGCCATTAAGGACATGCACGAAGAGTATGCAGATAAGGAc                                                                                                                                                                                                                              <  1:113100/299‑1 (MQ=255)`  
                                                                                 `cGGCAAGAGAAAAAAAGGAATAAAACTATAGGAGAAATTATTATGGCTATGACAAAGAAATTTAAAGGGTCCTTCGACGTTACCGCAAAGATGTCGTCTGACGTTCAGGCAATCTTAGAGAAAGATATGCTGCATCTATGTAAGCAGGTCGGCTCAGGTGCGAGTGTCCCCAATGGTAAACAGAAGGAAATGATTGTCCAGTTCCTGACACACGGTATGGAAGGATTGATGACATTCGTAGTACGTACATCATTTCGTGAGGCCATTAAGGACATGCACGAAGAGTATGCAGATAAGGAct                                                                                                                                                                                                                             <  1:330557/301‑1 (MQ=255)`  
                                                                                       `gagaAACAAAGGCATAAAACTATAGGAGAAATTATTATGGCTATGACAAAGAAATTTAAAGTGTCCTTCGACGTTACCGCAAAGATGTCGTCTGACGTTCAGGCAATCTTAGAGAAAGATATGCTGCATCTATGTAAGCAGGTCGTCTCAGGTGCGATTGTCCCCAATGGTAAACAGCAGGAAATGATTGTCCAGTTCCTGACACACGATATGGAAGGATTGATGACATTCGTAGTACGTACATCATTTCGTGAGGCCATTAAGGACATGCACGAtgagctgtctcttatacacatctccc                                                                                                                                                                                                                       >  1:63706/1‑279 (MQ=255)`  
                                                                                          `gtgggctcggagatgtgtataAGAGACAGTATTATGGCTATGACAAAGAAATTTAAAGTGTCCTTCGACGTTACCGCAAAGATGTCGTCTGACGTTCAGGCAATCTTAGAGAAAGATATGCTGCATCTATGTAAGCAGGTCGGCTCAGGTGCGATTGTCCCCAATGGTAAACAGAAGGAAATGATTGTCCAGTTCCTGACACACGGTATGGAAGGATTGATGACATTCGTAGTACGTACATCATTTCGTGAGGCCATTAAGGACATGCACGAAGAGTATGCAGATAAGGACTCTTTCAAAc                                                                                                                                                                                                                    <  1:365457/284‑1 (MQ=255)`  
                                                                                          `agagacaGGCATAAAACTATAGGAGAAATTATTATGGCTATGACAAAGAAATTTAAAGTGTCCTTCGACGTTACCGCAAAGATGTCGTCTGACGTTCAGGCAATCTTAGAGAAAGATATGCTGCATCTATGTAAGCAGGTCGGCTCAGGTGCGATTGTCCCCAATGGTAAACAGAAGGAAATGATTGTCCAGTTCCTGACACACGGTATGGAAGGATTGATGACATTCGTAGTACGTACATCATTTCGTGAGGCCATTAAGGACATGCACAAAGAGTATGCAGATAAGGACTCTTTCAAAc                                                                                                                                                                                                                    <  1:479098/295‑1 (MQ=255)`  
                                                                                          `aaaaaaAGGCAGAAAACTATAGGAGAAAGAATTATGGCAATGACAAAGAAATTTAAAGTGAACTTCGACGTTACCGCAAAGATGTCGTCTGACGTGCAGGAAATCTTAGAGAAAGATAAGATGCAGTGATGTAAGCATGTCGGTTCAGGTGCGATGGTCCTCAATGGTAAACAGAAGGAAAGGATTGTCCAGTTCGTGACAAACGAGATGGAAGGATTGATGACATGCGTAGTACGTACATCATTTCGTGAGGCCATTAAGGACATGCACGAAGAGTATGCAGATAAGGACTCTTTCAAAc                                                                                                                                                                                                                    <  1:70882/301‑1 (MQ=255)`  
                                                                                          `aaaCAAAGGCATAAAACTATAGGAGAAATTATTATGGCTATGACAAAGAAATTTAACGTGTCCTTCGACGTTACCGCAAAGATGTCGTCTGACGTTCAGGCAATCTTAGAGAAGGATATGCTGCATCTATGTAAGCAGGTCGGCTCAGGTGCGATTGTCCCCAATGGTAAACAGAAGGAAATGATTGTCCAGTTCCTGACACACGGTATGGAAGGATTGATGACATTCGTAGTACGTACATCATTTCGTGAGGCCATTAAGTACATGCACGAAGAGTATGCAgctgtctcttatacacatc                                                                                                                                                                                                                    >  1:31092/1‑283 (MQ=255)`  
                                                                                                  `gCATAAAACTATAGGAGAAATTATTATGGCTATGACAAAGAAATTTAAAGTGTCCTTCGACGTTACCGCAAAGATGTCGTCTGACGTTCAGGCAATCTTAGAGAAAGATATGCTGCATCTATGTAAGCAGGTCGGCTCAGGTGCGATTGTCCCCAATGGTAAACAGAAGGAAATGATTGTCCAGTTCCTGACACACGGTATGGAAGGATTGATGACATTCGTAGTCAGTACATAATTTCGTGAGGCCATTAAGGACATGCACGAAGAGTATGCAGATAAGGACTCTTTCAAACACTCTCCt                                                                                                                                                                                                            >  1:141547/1‑301 (MQ=255)`  
                                                                                                  `gCATAAAACTATAGGAGAAATTATTATGGCTATGACAAAGAAATTTAAAGTGTCCTTCGACGTTACCGCAAAGATGTCGTCTGACGTTCAGGCAATCTTAGAGAAAGATATGCTGCATCTATGTAAGCAGGTCGGCTCAGGTGCGATTGTCCCCAATGGTAAACAGAAGGAAATGATTGTCCAGTTCCTGACACACGGTATGGAAGGATTGATGACATTCGTAGTACGTACATCATTTCGTGAGGCCATTAAGGACATGCACGAAGAGTATGCAGATAAGGACTCTTTCAAACAATCTCCt                                                                                                                                                                                                            >  1:195569/1‑301 (MQ=255)`  
                                                                                                       `agaCTATAGGAGAAATTATTATGGCTATGACAAAGAAATTTAAAGTGTCCTTCGACGTTACCGCAAAGATGTCGTCTGACGTTCAGGCAATCTTAGAGAAAGATATGCTGCATCTATGTAAGCAGGTCGGCTCAGGTGCGATTGTCCCCAATGGTAAACAGAAGGAAATGATTGTCCAGTTCCTGACACACGGTATGGAAGGATTGATGACATTCGTAGTACGTACATCATTTCGTGAGGCCATTAAGGACATGCACGAAGAGTATGCAGATAAGGACTCTTTCAAACAATCTCCTGCAAc                                                                                                                                                                                                       <  1:361325/299‑1 (MQ=255)`  
                                                                                                         `aCTATAGGAGAAATTATTATGGCTATGACAAAGAAATTTAAAGTGTCCTTCGACGTTACCGCAAAGATGTCGTCTGACGTTCAGGCAATCTTAGAGAAAGATATGCTGCATCTATTTAAGCAGGTCGGCTCAGGTGCGATTGTCCCCAATGGTAAACAGCAGGAAATGATTGTCCAGTTCCTGACCCACGGTATGGAAGGATTGATGACATTCGTAGTACGTACATCATTTCGTGAGGACATTAAGGACCTGCACGAAGAGTATGCAGATAAGGACTCTTTCACACAATCTCCTGAACCAg                                                                                                                                                                                                     >  1:285205/1‑301 (MQ=255)`  
                                                                                                          `cTATAGGAGAAATTATTATGGCTATGACAAAGAAATTTAAAGTGTCCTTCGACGTTACCGCAAAGAAGTCGTCTGACGTTCAGGCAATCTTAGAGAAAGATATGATGCATCTATGTAAGCAGGTCGGCTCAGGTGCGATTGTCCCCAATGGTAAACAGAAGGAAATGATTGTCCAGTTCCTGACACACGGTATGGAAGGATTGATGACATTCGTAGTACGTACATCATTTCGTGAGGCCATTAAGGACATGCACGAAGAGTATGAAGATAAGGACTCTTTCAAACAATCTCCTGCAACAGt                                                                                                                                                                                                    >  1:297494/1‑301 (MQ=255)`  
                                                                                                               `ggAGAAATTATTATGGCTATGACAAAGAAATTTAAAGTGTCCTTCGACGTTACCGCAAAGATGTCTTCTGACGTTCAGGCAATCTTAGAGAAAGATATGCTGCATCTATGTAAGCAGGTCGGCTCAGGTGCGATTGTCCCCAATGGTAAACAGAAGGAAATGATTGTCCAGTTCCTGACACACGGTATGGAAGGATTGATGACATTCGTAGTACGTACATCATTTCGTGAGGCCATTAAGGACATGCACGAAAAGTATGCAGATAAGGACTCTTTCAAACAATCTCCTACAACCATACggg                                                                                                                                                                                               >  1:81834/1‑301 (MQ=255)`  
                                                                                                                 `agaAAGTATTATGGCTATGACAAAGAAATTTAAAGTGTCCTTCGACGTTACCGCAAAGATGTCGTCTGACGTTCAGGCAATCTTAGAGAAAGATATGCTGCATCTATGTAAGCAGGTCGGCTCAGGTTCGATTGTCCCCAATGGTAAACAGAAGGAAATGATTGTCCAGTTCCTGACACACGGTATGGAAGGATTGATGACATTCGTAGTACGTACATCATTTCGTGAGGCCATTAAGGACATGCACGAAGAGTATGCAGATAAGGACTCTTTCAAACAATCTCCTGCAACAGTACGGGAg                                                                                                                                                                                             <  1:166722/301‑1 (MQ=255)`  
                                                                                                                  `taaaTTATTATGGCTATGACAAAGAATTTTAAAGTGTCCTTCGACGTTACCGCAAAGATGTCGTCTGACGTTCATGCAATCTTAGAGAAAGATATGCTGCATCTATGTAAGCAGGTTGGCTCAGGTGCGATTGTCCCCAATGGTAAACAGAAGGAAATGATTGTCCAGTTCCTGACACACGGTATGGAAGGATTGATGACATTCGTAGTACGTACATCATTTCGTGAGGCCATTAAGGACATGCACGAAGAGTATGCAGATAAGGACTCTTTCAAACAATCTCCTGCAACAGTACGGGAgg                                                                                                                                                                                            <  1:340094/300‑1 (MQ=255)`  
                                                                                                                  `gaAATTATTATGGCTATGACAAAGAAATTTAAAGTGTCCTTCGACGTTACCGCAAAGATGTCGTCTGACGTTCAGGCAATCTTAGAGAAAGATATGCTGCATCTATGTAAGCAGGTCGGCTCAGGTGCGATTGTCCCCAATGGTAAACCGAAGGAAATGATTGTCCAGTTCCTGACACACGGTATGGAAGGATTGATGACATTCGTAGTACGTACATCATTTCGTGAGGCCATTAAGGACATGCACGAAGAGTATGCCGATAAGGACTGTTTCAAACAATCTCCTGCAACAGTACGGGAgg                                                                                                                                                                                            >  1:87779/1‑301 (MQ=255)`  
                                                                                                                  `gaAATTATTATGGCTATGACAAAGAAATTTAAAGATTCCATCGACGTTACCGCAAAGATGTCGTCTGACGGTCAGGCAATCATAGAGAAAGCTATGCTGCATCTATGTAAGCAGGTCGACTCAGGTGCGATTGTCCCAAATGGTAATCAGAAGGAAATGATTGTCCAGTTCCTGACACACGGTATGGAAGGATTGATGACATTCGTAGTACGTACATCATTTCGTGATGCCATTAAGGACATGCACGAAGAGTATGCAGATAAGGACTCTTTCGAACAATCTCCTGCAACAGTACGGGAgg                                                                                                                                                                                            <  1:120807/301‑1 (MQ=255)`  
                                                                                                                  `aaaaTTAGTATGGCAATGACAAAGAAAGTTAAAGTGTCCTTCGACGTTACCGCAAAGATGTCGTATGACGTTCAGGCAATCTTAGAGAAAGATATGCTGCATCTATGTAAGCAGGTCGGCTCAGGTGCGATTGTACCCAATGGTAAACAGAAGGAAATGATTGTCCAGTTCCTGACACAAGGTATGGAAGGATTGATGACATTCGTAGTACGTACATCATTTCGTGAGGACATTAAGGACATGCACGAAGAGTATGCAGATAAGGACTCTTTCAAACAATCTCCTGCAACAGTACGGGAgg                                                                                                                                                                                            <  1:144519/300‑1 (MQ=255)`  
                                                                                                                          `cagggCTATGACAAAGAAATTTAAAGTGTCCTTCGACGTTACCGCAAAGATGTCGTCTGACGTTCAGGCAATCTTAGAGAAAGATATGCTGCATCTATGTAAGCAGGTCGGCTCAGGTGCCATTGTCCCCAATGGTAAACAGAATGAAATGATTGTCCAGTTCCTGACACACGGTATGGAAGGATTGATGACATTAGTAGTACGTACATCATTTCGTGAGGCCATTAAGGACATGCACGAAGAGTATGCAGATAAGGACTCTTTCAAACAATCTCCTGCAACAGTACGGGAGGTGTTCTGa                                                                                                                                                                                    <  1:414091/298‑1 (MQ=255)`  
                                                                                                                            `tGGCTATGACAAAGAAATTTAAAGTGTCCTTCGACGTTACCGCAAAGATGTCGTCTGACGTTCAGGCAATCTTAGAGAAAGATATGCTGCATCTATGTAAGCAGGTCGGCTCAGGTGCGATTGTCCCCAATGGTAAACAGAAGGAAATGATTGTCCAGTTCCTGACACACGGTATGGAAGGATTGATGACATTCGTAGTACGTACATCATTTCGTGAGGCCATTAAGGACATGCACGAAGAGTATGCAGATAAGGACTCTTTCAAACAATCTCCTGCAACAGTACGGGAGGTGTTCTGATg                                                                                                                                                                                  <  1:280222/301‑1 (MQ=255)`  
                                                                                                                            `tGGCTATGAACAAGAAAATTAAAGGGGCCTTCGACGTTACCCCAAAGATGTCGTCTGACGTTCAGGCAATCTTAGAGAAAGATATGCTGCATCTATGTAAGCAGGTCGGCTCAGGTGCGATTGTCCCCAATGGTAAACAGAAGGAAATGATTGTCCAGTTCCTGACACACGGTATGGAAGGATTGATGACATTCGTAGTACTTACATCATTTCGTGAGGCCATTAAGGACATGCACGAAGAGTATGCAGATAAGGACTCTTTCAAACAAACTCCTGCAACAGTACGGGAGGTGTTCTGATg                                                                                                                                                                                  <  1:336229/301‑1 (MQ=255)`  
                                                                                                                             `ggCTATGACAAAGAAATTTAAAGTGTCCTTCGACGTTACCGCAAAGATGTCGTCTGACGTTCAGGCAATCTTAGAGAAAGATATGCTGCATCTATGTAAGCAGGTCGGCTCAGGTTCGATTGTCCCCAATGGTAAACAGAAGGAAATGATTGTCCAGTTCCTGACACACGGTATGGAAGGATTGATGACATTCGTAGTACGTACATCATTTCGTGACGCCATTAAGGACATGCACGAAGAGTATGCAGATAAGGACTCTTTCAAACAATCTCCTGCAACAGTACGGGAGctgtctcttata                                                                                                                                                                                 >  1:210645/1‑293 (MQ=255)`  
                                                                                                                             `ggCTATGACAAAGAAATTTAAAGTGTCCTTCGACGTTACCGCAAAGATGTCGTCTGACGTTCAGGCAATCTTAGAGAAAGATATGCTGCATCTATGTAAGCAGGTCGGCTCAGGTGCGATTGTCCCCAATGGTAAACAGAAGGAAATGATTGTCCAGTTCCTGACACACGGTATGGAAGGATTGATGACATTCGTAGTACGTACATCATTTCGTGAGGCCATTAAGGACATGCACGAAGAGTATGCAGATAAGGACTCTTTCAAACAATCTCCTGCAACAGTACGGGAGGTGTTCTGATgc                                                                                                                                                                                 >  1:185827/1‑300 (MQ=255)`  
                                                                                                                             `ggCTATGACAAAGAAATTTAAAGTGTCCTTCAACGTTACCGCAAATATGTCGTCTGACGTTCAGGCAATCTTAGAGAAAGATATGCTGCATCTATGTACGCAGGTCGGCTCAGGTGCGATTGTCCCCAATGGTAAACAGAAGGAAATGATTGTACAGTGCCTGACACACGGTATGGAAGGATTGATGACATTCGTAGTACGTACCTCATTTCGTGCGGCCATTAATGACATGCACGACAAGTCTGCAGATAAGAACACTTTCAAACAATTTCCTGCAAACGTCCGGGAGgttgtttgatat                                                                                                                                                                                 >  1:252760/1‑291 (MQ=255)`  
                                                                                                                                   `aggtaaagaaTTTAAAGTGTCAGTCGACGTTCCCGCAAAGAGGTCGTCTGACGTTCAGGAAATCTTAGAGAAAGATATGCTGCATCTATGTAAGCAGGTCGGTTCAGGTGCGATTGTCCCCAATGGTAAACAGAAGGAAATGATTGTCAAGTTCCTGACATACGGTATGGAAGGATTGATGACATTCGTAGTACGTACATCATTTCGTGAGGCCATTAAGGACATGCACGAAGAGTATGCAGATAAGGACTCTTTCAAACAATCTCCTGCAACAGTACGGGAGGTGTTCTGATGTCTGACt                                                                                                                                                                           <  1:325356/293‑1 (MQ=255)`  
                                                                                                                                   `agagacagaaTTTAAAGGGTCCTTCGAAGTTACCGCAAAGATGTCGTCTGACGTTCAGGCAATCTTAGAGAAAGATATGCTGCATCTATGTAAGCAGGTCGGCTCAGGTGCGATTGTCCCCAATGGTAAACAGAAGGAAATGATTGTCCAGTTCCTGACACACGGTATGGAAGGATTGATGACATTCGTAGTACGTACATCATTTCGTGAGGCCATTAAGGACATGCACGAAGAGTATGCAGATAAGGACTCTTTCAAACAATCTCCTGCAACAGTACGGGAGGTGTTCTGATGTCTGACt                                                                                                                                                                           <  1:100039/293‑1 (MQ=255)`  
                                                                                                                                            `acttAAAGTGTCCTTCTACGTTACCGCAAAGATAGCGTATGACGTTCAGGCAATCTTAGAGAAAGATATGCTGCGTCTGTATAAGCAGGTCGGCTCAGGTGCGATTGTCCCCAATGGTAAACAGAAGGAAATGATTGTCCAGTTCCTGACACACGGTATGGTAGGATTGATGCCATTCGTAGTACGTACATCATTTCGTGAGGCCATTAAGGACATGCACGAAGAGTATGCAGATAAGGACTCTTTCAAACATTCTCCTGCAACAGTACGGGAGGTGTTCTGATGTCTGACTACCTGAAAg                                                                                                                                                                  <  1:474374/299‑1 (MQ=255)`  
                                                                                                                                                                           `aTGTCGGCTGACGTTCAGGCAATCTTAGAGAAAGATATGCTGCATCTATGTAAGAAGGTCGGCTCGGGTGCGATTGTCCCCAATGGTAAACAGAAGGAAATGATTGTCCAGTTCCTGACACACGGTATGGAAGGATTGATGACATTCGTAGTACGTACATCATTTCGTGAGGCCATTAAGGACATGCACGAAGAGTATGCAGATAAGGACTCTTTCAAACAATCTCCTGCAACAGTACGGGAGGTGTTCTGATGTCTGACTACCTGAAAGTGCTGCAAGCAATCAAAAGTTGCCCTAAGAc                                                                                                                                   <  1:3736/301‑1 (MQ=255)`  
                                                                                                                                                                                     `gcGTTAAGGCAATCTTAGAGAAAGATATGCTGCATCTATGTAAGCAGGTCGGCTCAGGTGCGATTGTCCACAATGGTAAACAGAAGGAAATGATTGTCCAGTTCCTGACACACGGTATGGAAGGATTGATGACATTCGTAGTACGTACATCATTTCGTGAGGCCATTAAGGACATGCACGAAGAGTATGCAGATAAGGACTCTTTCAAACAATCTCCTGCAACAGTACGGGAGGTGTTCTGATGTCTGACTACCTGAAAGTGCTGCAAGCAATCAAAAGTTGCTCTAAGACTTTCCAGTcc                                                                                                                         <  1:315836/300‑1 (MQ=255)`  
                                                                                                                                                                                      `cGTTCAGGCAATCTTAGAGAAAGATATGCTGCATCTATGTAAGCAGGTCGGCT‑AGGTGCGATTGTCCCCAATGGTAAACAGAAGGAAATGATTGTCCAGTTCCTGACACACGGTATGGAAGGATTGATGACATTCGTCGTACGTACATCATTTCGTGAGGCCATTAAGGACATGCACGAAGAGTATGCAGATAAGGACTCTTTCAAACAATCTCCTGCAACAGTACGGGAGGTGTTCTGATGTCTGACTACCTGAACGTGCTGCAAGCAATCAAAAGTTGCCCTAAGACTTTCCAGTCCaa                                                                                                                       >  1:242101/1‑301 (MQ=255)`  
                                                                                                                                                                                                  `cTTATAGAAAGTCATGCTGGATCTATGTAAGCAGGTCGGCGCAGGTGGGATTGTCCCCAATGGTAAAAAGAAGGAAATGATTGTCCCGTTCCTGACACCCGGTATTGAAGGATTGATGACATTCGTAGTACGTACATCATTTCGTGAGGCCATTAAGGACATGCACGAAGAGTATGCAGATAAGGACTCTTTCAAACAATCTCCTGCAACAGTACGGGAGGTGTTCTGATGTCTGACTACCTGAAAGTGCTGCAAGCAATCAAAAGTTGCCCTAAGACTTTCCAGTCCAACTATGTACGGa                                                                                                            <  1:491484/301‑1 (MQ=255)`  
                                                                                                                                                                                                    `tAGAGAAAGATATGCTGCATCTATGTAAGCAGGTCGGCTCAGGTGCGATTGTCCCCAATGGTAAACAGAAGGAAAGGATTGTCCAGTTCCTGACACACGGTATGGAAGGATTGATGACATTCGTAGTACGTACATCATTTCGTGAGGCCATTAAGGACATGCACGAAGAGTATGCAGATAAGGACTCTTTCAAACAATCTCCTGCAACAGTACGGGAGGTGTTCTGATGTCTGACTACCTGAAAGTGCTGCAAGCAATCAAAAGTTGCCCTAAGACTTTCCAGTCCAACTATGTACGGAAc                                                                                                          <  1:265841/301‑1 (MQ=255)`  
                                                                                                                                                                                                    `tAGAGAAAGATATGCTGCATCTATGTAAGCAGGTCGGCGCAGGTGCGATTGTCCCCAATGGTAAACAGAAGGAAATGATTGTCCAGTTCCTGACACACGGTATGGAAGGATTGATGACATTCGTAGTACGTACATCATTTCGTGAGGCCATTAAGGACATGCACGAAGAGTATGCAGATAAGGACTCTTTCAAACAATCTACTGCAACAGTACGGGAGGTGTTCTGATGTCTGACTACCTGAAAGTGCTGCAAGCAATCAAAAGTTGCCCTAAGACTTTCCAGTCCAACTATGTACGGAAc                                                                                                          <  1:444636/301‑1 (MQ=255)`  
                                                                                                                                                                                                         `aagagacggctgcATCTATGTAAGCAGGTCGGCTCAGGTGCGATTGTCCCCAATGGTAAACAGAAGGAAATGATTGTCCAGTTCCTGACACACGGTATGGAAGTATTGATGACATTCGTAGTACGTACATCATTTCGTGAGGCCATTAAGGACATGCACGAAGAGTATGCAGATAAGGACTCTTTCAAACAATCTCCTGCAACAGTACGGGAGGTGTTCTGATGTCTGACTACCTGAAAGTGCTGCAAGCAATCAAAAGTTTCCCTAAGACTTTCCAGTCCAACTATGTACGGAACAATGc                                                                                                     <  1:91432/293‑1 (MQ=255)`  
                                                                                                                                                                                                            `gATATGCTGCATCTATGTAAGCAGGTCGGCTCAGGTGCGATTGTCCCCAATGGTAAACAGAAGGAAATGATTGTCCAGTTCCTGACACACGGTATGGAAGGATTGATGACATTCGTAGTACGTACATCATTTCGTGAGGCCATTAAGGACATGCACGAAGAGTATGCAGATAAGGACTCTTTCAAACAATCTCCTGCAACAGTACGGGAGGTGTTCTGATGTCTGACTACCTGAAAGTGCTGCAAGCAATCAAAAGTTGCCCTAAGACTTTCCAGTCCAACTATGTACGGACCAATGCGac                                                                                                  >  1:153291/1‑300 (MQ=255)`  
                                                                                                                                                                                                                       `tCTATGTAAGCAGGTCGGCTCAGGTGCGATTGTCCCCAATGGTAAACAGAAGGAAATGATTGTCCAGTTCCTGACACACGGTATGGAAGGATTGATGACATTCGTAGTACGTACATCATTTCGTGAGGCCATTAAGGACATGCACGAAGAGTATGCAGATAAGGACTCTTTCAAACAATCTCCTGCAACAGTACGGGAGGTGTTCTGATGTCTGACTACCTGAAAGTGCTGCAAGCAATCAAAAGTTGCCCTAAGACTTTCCAGTCCAACTATGTACGGAACAATGCGAGCCTCGTAGCgg                                                                                       <  1:483893/301‑1 (MQ=255)`  
                                                                                                                                                                                                                        `cTATGTAAGCAGGTCGGCTCAGGTGCGATTGTCCCCAATGGTAAACAGAAGGAAATGATTGTCCAGTTCCTGACACACGGTATGGAAGGATTGATGACATTCGTAGTACGTACATCATTTCGTGAGGCCATTAAGGACATGCACGAAGAGTATGCAGATAAGGACTCTTTCAAACAATCTCCTGCAACAGTACGGGAGGTGTTCTGATGTCTGACTACCTGAAAGTGCTGCAAGCAATCAAAAGTTGCCCTAAGACTTTCCAGTCCAACTATGTACGGAACAATGCGAGCCTCGTAGCggg                                                                                      >  1:394498/1‑300 (MQ=255)`  
                                                                                                                                                                                                                         `tATGTAAGCAGGTCGGCTCAGGTGCGATTGTCCCCAATGGTAAACAGAAGGAAATGATTGTCCAGTTCCTGACACACGGTATGGAAGGATTGATGACATTCGTAGTACGTACATCATTTCGTGAGGCCATTAAGGACATGCACGAAGAGTATGCAGATAAGGACTCTTTCAAACAATCTCCTGCAACAGTACGGGAGGTGTTCTGATGTCTGACTACCTGAAAGTGCTGCAAGCAATCAAAAGTTGCCCTAAGACTTTCCAGTCCAACTATGTACGGAACAATGCGAGCCTCGTAGCGGAg                                                                                     >  1:409453/1‑301 (MQ=255)`  
                                                                                                                                                                                                                            `gTAAGCAGGTCGGCTCAGGTGCGATTGTCCCCAATGGTAAACAGAAGGAAATGATTGTCCAGTTCCTGACACACGGTATGGAAGGATTGATGACATTCGTAGTACGTACATCATTTCGTGAGGCCATTAAGGACATGCACGAAGAGTATGCAGATAAGGACTCTTTCAAACAATCTCCTGCAACAGTACGGGAGGTGTTCTGATGTCTGACTACCTGAAAGTGCTGCAAGCAATCAAAAGTTGCCCTAAGACTTTCCAGTCCAACTATGTACGGAACAATACGAGCCTAGTAGCGGAGGcc                                                                                  >  1:67664/1‑301 (MQ=255)`  
                                                                                                                                                                                                                                              `gTGCGATTGTCCCCAATGGTAAACAGAAGGTAATGATTGTCCAGTTCCTGACACACGGTATGGAAGGATTGATGACATTCGTAGTACGTACATCATTTCGTGAGGCCATTAAGGACATGCACGAAGAGTATGCAGATAAGGACTCTTTCAAACAATCTCCTGCAACAGTACGGGAGGTGTTCTGATGTCTGACTACCTGAAAGTGCTGCAAGCAATCAAAAGTTGCCCTAAGACTTTCCAGACCAACTATGTACGGAACAATGCGAGCCTCGTAGCGGAGGCCGCTTCCTGtctcttatac                                                                >  1:59285/1‑292 (MQ=255)`  
                                                                                                                                                                                                                                              `gTGCGATTGTCCCCAATGGTAAACAGAAGGAAATGATTGTCCAGTTCCTGACACACGGTATGGAAGGATTGATGACATTCGTAGTACGTACATCATTTCGTGAGGCCATTAAGGACATGCACGAAGAGTATGCAGATAAGGACTCTTTCAAACAATCTCCTGCAACAGTACGGGAGGTGTTCTGATGTCTGACTACCTGAAAGTGCTGCAAGCAATCAAAAGTTGCCCTAAGACTTTCCAGTCCAACTATGTACGGAACAATGCGAGCCTCGTAGCGGAGGCCGCTTCCCGTGGTCACAtc                                                                <  1:496831/301‑1 (MQ=255)`  
                                                                                                                                                                                                                                               `tgtataagagacagaTGGTAAACAGAAGGAAATGATTGTCCAGTTCCTGACAAACGGTATGGAAGGATTGATGACATTCGTAGTACGTACATCATTTCGTGAGGCCATTAAGGACATGCACGAAGAGTATGCAGATAAGGACTCTTTCAAACAATCTCCTGCAACAGTACGGGAGGTGTTCTGATGTCTAACTACCTGAAAGTGCTGCAAGCAATCAAAAGTTGCCCTAAGACTTTCCAGTCCAACTATGTACGGAACAATGCGAGCCTCGTAGCGGAGGCCGCTTCCCGTGGTCACAtct                                                               <  1:441907/287‑1 (MQ=255)`  
                                                                                                                                                                                                                                                       `tCCCCAATGGTAAACAGAAGGAAATGATTGTCCAGTTCCTGACACACGGTATGGAATGATTGATGACATTCGTAGTACGTACATCATTTCGTGAGGCCATTAAGGACATGCACGAAGAGTATGCAGATAAGGACTCTTTCAAACAATCTCCTGCAACAGTACGGGAGGTGTTCTGATGTCTGACTACCTTAAAGTGCTGCAAGCAATCAAAAGTTGCCCTAAGACTTTCCAGTCCAACTATGTACGGAACAATGCGAGCCTCGTAGCGGAGGCCGCTTCCCGTGGTCACATCTCGTGCCTg                                                       <  1:117277/301‑1 (MQ=255)`  
                                                                                                                                                                                                                                                            `tgtataagagacagggAAATGATTGTCCAGTTCCTGACACACGGTATGGAAGGATTGATGACATTCGTAGTACGTACATCATTTCGTGAGGCCATTAAGGACATGCACGAAGAGTATGCAGATAAGGACTCTTTCAAACAATCTCCTGCAACAGTACGGGAGGTGTTCTGATGTCTGACTACCTGAAAGTGCTGCAAGCAATCAAAAGTTGCCCTAAGACTTTCCAGTCCAACTATGTACGGAACAATGCGAGCCTCGTAGCGGAGGCCGCTTCCCGTGGTCACATCTCGTGCCTGactac                                                  <  1:202160/287‑1 (MQ=255)`  
                                                                                                                                                                                                                                                             `aTGGTAAACAGAAGGAAATGATTGTCCAGTTCCTGACACACGGTATGGAAGGATTGATGACATTCGTAGTACGTACATCATTTCGTGAGGCCATTAAGGACATGCACGAAGAGTATGCAGATAAGGACTCTTTCAAACAATCTCCTGCAACAGTACGGGAGGTGTTCTGATGTCTGACTACCTGAAAGTGCTGCAAGCAATCAAAAGTTGCCCTAAGAATTTCCAGTCCAACTATGTACGGAACAATGCGAGCCTCGTAGCGGAGGCCGATTACCGTGGTCACATCTCGTGCCTGactact                                                 >  1:236810/1‑301 (MQ=255)`  
                                                                                                                                                                                                                                                             `aTGGTAAACAGAAGGAAATGATTGTCCAGTTCCTGACACACGGTATGGAAGGATTGATGACATTAGTAGTACGTACATCATTTCGTGAGGCCATTAAGGACATGCACGAAGAGTATGCAGATAAGGACTCTTTCAAACAATCTCCTGCAACAGTACGGGAGGTGTTATGATGTCTGACTACCTGAAAGTGCTGCAAGCAATAAAAAGTTGCCCTAAGACTTTCCAGTCCAACTATATACGGAACAATGCGAGCCTCGTAGCGGAGGCCGCTTCCCGTGGTCACATCTCGTGCCTGactact                                                 >  1:128599/1‑301 (MQ=255)`  
                                                                                                                                                                                                                                                                 `tgtgtataagagacagATTGTCCAGTTCCTGACACACGGTATGGAAGGATTGATGACATTCGTAGTACGTACATCATTTCGTGAGGCCATTAAGGACATGCACGAATAGTATGCAGATAAGGACTCTTTCAAACAATCTCCTGCAACAGTACGGGAGGTGTTCTGATGTCTGACTACCTGAAAGTGCTGCAAGCAATCAAAAGTTGCCCTAAGACTTTCCAGTCCAACTATGTACGGAACAATGCGAGCCTCGTAGCGGAGGCCGCTTCCCGTGGTCACATCTCGTGCCTGACTACTAGTg                                             <  1:275707/286‑1 (MQ=255)`  
                                                                                                                                                                                                                                                                 `tAAACAGTAGGAAATGATTGTCCAGTTCCTGACACTCGGTATGGAAGGATTGATGACATTTGTAGTACGTACATCATTTCGTGAGGCCAATAAGGACATGCAAGAAGAGTATGCAGATAAGGATTCTTTCAAACAATCTCCTGCAACAGTACGGGGTGTGTTCTGATGTCTGACTACCTGAAAGTGCTGCAAGCAATCAAAAGTTGCCCTAAGACTTTCCAGTCCAACGATGTACGGAACAATGCGAGCCTCGTAGCGGAGGCCGCTTCCCGTGGGCACATCTCTTGCCTGACTACTAGTg                                             <  1:506283/301‑1 (MQ=255)`  
                                                                                                                                                                                                                                                                 `tAAACAGAAGGAAATTATTGTCCAGTTCCTGACACACGGTATGGAAGGATTGATGACATTCGTAGTACGTACATCATTTCGTGAGGCCATTAAGGACATGCACGAAGAGTATGCAGATAAGGACTCTTTCAAACAATCTCCTGCAACAGTACGGGAGGTGTTCTGATGTCTGACTACCTGAAAGTGCTGCAAGCAATCAAAAGTTTCCCTAAGACTTTCCAGTCCAACTATGTACGGAACAATGCGAGCCTCGTAGCGGAGGCCGCTTCCCGTGGTCACATCTCGTGCCTGACTACTAGTg                                             <  1:174015/301‑1 (MQ=255)`  
                                                                                                                                                                                                                                                                 `ggagatgtgtataagagacagccAGTTCCTGAAACACGGTATGGAAGGATTGATGACATTCGTAGTACGTACATCATTTCGTGATGCCATTAAGGACATGCACGAAGAGTATGCAGATAATGACTCTTTCAAACAATCTCCTGCAACAGTACGGGAGGTGTTCTGATGTCTGACTACCTGAAAGTGCTGCAAGCAATCAAAAGTTGCCCTAAGACTTTCCAGTCCAACTATGTACGGAACAATGCGAGCCTCGTAGCGGAGGCCGCTTCCCGTGGTCACATCTCGTGCCTGACTACTAGTg                                             <  1:383015/280‑1 (MQ=255)`  
                                                                                                                                                                                                                                                                     `ccggagatgtgtataagagacagccTGACACCCGGTATGGAAGGATTGATGACATTCGTAGTACGTACATCATTTCGTGAGGCCATTAAGGACATGCACGAAGAGTATGCAGATAAGGACTCTTTCAAACAATCTCCTGCAACAGTACGGGAGGTGTTCTGATGTCTGACTACCTGAAAGTGCTGCAAGCAATCAAAAGTTGCCCTAAGACTTTCCAGTCCAACTATGTACGGAACAATGCGAGCCTCGTAGCGGAGGCCGCTTCCCGTGGTCACATCTCGTGCCTGACTACTAGTGGACg                                         <  1:206115/278‑1 (MQ=255)`  
                                                                                                                                                                                                                                                                          `ggAAATGATTGTCCAGTTCCTGACACACGGTATGGAAGGATTGATGACATTCGTAGTACGTACATCATTTCGTGAGGCCATTAAGGACATGCACGAAGAGTATGCAGATAAGGACTCTTTCAAACAATCTCCTGCAACAGTACGGGAGGTGTTCTGATGTCTGACTACCTGAAAGTGCTGCAAGCAATCAAAAGTTGCCCTAAGACTTTCCAGTCCAACTATGTACGGAACAATGCGAGCCTCGTAGCGGAGGCCGCTTCCCGTGGTCACATCTCGTGCCTGACTACTAGTTGACGTAACg                                    >  1:108374/1‑301 (MQ=255)`  
                                                                                                                                                                                                                                                                           `gAAATGATTGTCCAGTTCCTGACACACGGTATGGAAGGATTGATGACATTCGTAGTACGTACATCATTTCGTGAGGCCATTAAGGACATGCACGAAGAGTATGCAGATAAGGACTCTTTCAAACAATCTCCTGCAACAGTACGGGAGGTGTTCTGATGTCTGACTACCTGAAAGTGCTGCAAGCAATCAAAAGTTGCCCTAAGACTTTCCAGTCCAACTATGTACGGAACAATGCGAGCCTCGTAGCGGAGGCCGCTTCCCGTGGTCACATCTCGTGCCTGACTACTAGTGGACGTAACgg                                   <  1:460385/301‑1 (MQ=255)`  
                                                                                                                                                                                                                                                                           `gAAATGATGGTCCAGTTCCTGACACACGGTATAGAAGGATGGATGACATTCGTAGTACGTACATCATTTCGTGAGGCCATTAAGGACATGCACGAAGAGTATGCAGATAAGGACTCTTTCAAACAATCTCCTGCAACAGTACGGGAGGTGTTCTGATGTCTGACTACCTGAAAGTGCTGCAAGCAATCAAAAGTTGCCCTAAGACTTGCCAGTCCAACTATGTACGGAACAATGCGAGCCTCGTAGCGTAGGCCGCTTCCCGTGGTCACATCTCGTGCCTGACTACTAGTGGACGTAACgg                                   <  1:162766/301‑1 (MQ=255)`  
                                                                                                                                                                                                                                                                               `taaTTGTCCAGTTCCTGACACACGGTATGGAAGGATTGATGACATTCGTAGTACGTACATCATTTCGTGAGGCCATTAAGGACATGCACGAAGAGTATGCAGATAAGGACTCTTTCAAACAATCTCCTGCAACAGTACGGGAGGTGTTCTGATGTATGACTACCTGTAAGTGCTGCAAGCAATCAAAAGTTGCCCTAAGACTTTCCAGTCCAACTATGTACGGAACAATGCGAGCCTCGTAGCGGAGGCCGCTTCCCGTGGTCACATCTCGTGCCTGACTACTAGTGGACGTAACGGTGgc                               >  1:133633/3‑301 (MQ=255)`  
                                                                                                                                                                                                                                                                                    `gTCCAGTTCCTTACACACGGTATGGAAGGATTGATGACATTCGTAGTACGTACATCATTTCGTGAGGCCATTAAGGACATGCACGAAGAGTATGCAGATAAGGACTCTTTCAAACAATCTCCTGCAACAGTACGGGAGGTGTTCTGATGTCTGACTACCTGAAAGTGCTGCAAGCAATCAAAAGTTGCCCTAAGACTTTCCAGTCCAACTATGTACGGAACAATGCGAGCCTCGTAGCGGAGGCCGCTTCCCGTGGTCACATCTCGTGCCTGACTACTATTGGACGTAACggctgtctctt                          >  1:423905/1‑292 (MQ=255)`  
                                                                                                                                                                                                                                                                                    `gTCCAGTTCCTGACACACGGTATGGAAGGATTGATGACATTCGTAGTACGTACATCATTTCGTGAGGCCATTAAGGACATGCACGAAGAGTATGCAGATAAGGACTCTTTCAAACAATCTCCTGCAACAGTACGGGAGGTGTTCTGATGTCTGACTACCTGAAAGTGCTGCAAGCAATCAAAAGTTGCCCTAAGACTTTCCAGTCCAACTATGTACGGAACAATGCGAGCCTCGTAGCGGAGGCCGCTTCCCGTGGTCACATCTCGTGCCTGACTACTAGTGGACGTAACGGTGGCGCTTg                          >  1:270083/1‑301 (MQ=255)`  
                                                                                                                                                                                                                                                                                         `gTTCCTGACACACGGTATGGAAGGATTGATGACATTCGTAGTACGTACATCATTTCGTGAGGCCATTAAGGACATGCACGAAGAGTATGCAGATAAGGACTCTTTCAAACAATCTCCTGCAACAGTACGGGAGGTGTTCTGATGTCTGACTACCTGAAAGTGCTGCAAGCAATCAAAAGTTGCCCTAAGACTTTCCAGTCAAACTATGTACGGAACAATGCGAGCCTCGTAGCGGAGGCCGCTTCCCGTGGTCACATCTCGTGCCTGACTCCTAGTGGACGTAACGGTTGCGCTTGGGaaa                     >  1:181547/1‑301 (MQ=255)`  
                                                                                                                                                                                                                                                                                         `gTTCCTGACACACGGTATGGAAGGATTGATGACATTCGTAGTACGTACATCATTTCGTGAGGCCATTAAGGACATGCACGAAGAGTATGCAGATAAGGACTCTTTCAAACAATCTCCTGCAACAGTACAGGAGGTGTTCTGATGTCTGACTACCTGAAAGTGCTGCAAGCAATCAAAAGTTGCCCTAATACTTTCCAGTCCAACTATGTACGGAACAATGCGAGCCTCGTAGCGGAGGCCGCTTCCCGTGGTCACATCTCGTGCCTGACTACTAGTGGACGTAACGGTGGCGCTTGGGaaa                     >  1:188318/1‑301 (MQ=255)`  
                                                                                                                                                                                                                                                                                             `ctgaaaacgggTATGGAAGGATTGATGACATTCGTAGTACGTACATCATTGCGTGAGGCCATTAAGGACATGCACGAAGAGTATGCAGATAAGGACTCTTTCAAACAATCTCCTGCAACAGTACGGGAGGTGTTCTGATGTCTGACTACCTGAAAGTGCTGCAAGCAATCAAAAGTTGCCCTAAGACTTTCCAGTCCAACTATGTACGGAACAATGCGAGCCTCGTAGCGGAGGCCGCTTCCCGTGGTCACATCTCGTGCCTGACTACTAGTGGACGTAACGGTGGCGCTTGGGAAATCAc                 <  1:96703/292‑1 (MQ=255)`  
                                                                                                                                                                                                                                                                                               `gACACACGGTATGGAAGGATTGATGACATTCGTAGTACGTACATCATTTCGTGAGGCCATTAAGGACATGCACGAAGAGTATGCAGATAAGGACTCTTTCAAACAATCTCCTGCAACAGTACGGGAGGTGTTCTGATGTCTGACTACCTGAAAGTGCTGCAAGCAATCAAAAGTTGCCCTAAGACTTTCCAGTCCAACTATGTACGGAACAATGCGAGCCTCGTAGCGGAGGCCGCTTCCCGTGGTCACATCTCGTGCCTGACTACTAGTGGACGTAACGGTGGGGCTTGGGAAATCACTg               >  1:1036/1‑301 (MQ=255)`  
                                                                                                                                                                                                                                                                                                     `cGGTATGGAAGGATTGATGACATTCGTAGTACGTACATCATTTCGTGAGGCCATTAAGGACATGCACGAAGAGTATGCAGATAAGGACTCTTTCAAACAATCTCCTGCAACAGTACGGGAGGTGTTCTGATGTCTGACTACCTGAAAGTGCTGCAAGCAATCAAAAGTTGCCCTAAGACTTTCCAGTCCAACTATGTACGGAACAATGCGAGCCTCGTAGCGGAGGCCGCTTCCCGTGGTCACTTCTCGTGCCTGACTACTAGTGGACGTAACGGTGGCGCTTGGGAAATCACTGCTTCCg         >  1:170224/1‑301 (MQ=255)`  
                                                                                                                                                                                                                                                                                                     `cGGTATGGAAGGATTGATGACATTCGTAGTACGTACATCATTTCGTGAGGACATTAAGGACATGCACGAAGAGTATGCAGATAAGGACTCTTTCAAACAATCTCCTGCAACAGTACGGGAGGTGTTCTGATGTCTGACTACCTGAAAGTGCTGCAAGCAATCAAAAGTTGCCCTAAGACTTTCCAGTCAAACTATGTACGGAACAATGCGAGCCTCGTAGCGGAGGCCGCTTCCCGTGGTCACATCTCGTGCCTGACTACTAGTGGACGTAACGGTGGCGCTTGGGAAATCACTGCTTCCg         >  1:454976/1‑301 (MQ=255)`  
                                                                                                                                                                                                                                                                                                     `cGGTATGGAAGGATTGATGACATTCGTAGTACGTACATCATTTAGTGAGGCCATTAAGGACATGCACGAAGAGTATGCAGATAAGGACTCTTTCAAACAATCTCCTGCAACAGTACGGGAGGTGTTCTGATGTCTGACTACCTGAAAGTGCTGCAAGCAATCAAAAGTTGCCCTAAGACTTTCCAGTCCAACTATGTACGGAACAATGCGAGCCTCGTAGCGGAGGCCGCTTCCCGTGGTCACATCTCGTGCCTGACTACTAGTGGACGTAACggctgtctcttatacacatctccgagcc         >  1:149333/1‑275 (MQ=255)`  
                                                                                                                                                                                                                                                                                                       `gTATGGAAGGATTGATGACATTCGTAGTACGTACATCATTTCGTGAGGCCATTAAGGACATGCACGAAGAGTATGCAGATAAGGACTCTTTCAAACAATCTCCTGCAACAGTACGGGAGGTGTTCTGATGTCTGACTACCTGAAAGTGCTGCAAGCAATCAAAAGTTGCCCTAAGACTTTCCAGTCCAACTATGTACGGAACAATGCGAGCCTCGTAGCGGAGGCCGCTTCCCGTGGTCACATCTCGTGCCTGACTACTAGTGGACGTAACGGTGGCGCGTTGGAAATCACTGCTTCCGGt       >  1:247007/1‑301 (MQ=255)`  
                                                                                                                                                                                                                                                                                                       `gTATGGAAGGATTGATGACATTCGTAGTACGTACATCATTGCGTGAGGCCATTAAGGACATGCACGAAGAGTATGCAGATAAGGACTCTTTCAAACAATCTCCTGCAACAGTACGGGAGGTGTTCTGATGTCTGACTACCTGAAAGTGCTGCAAGCAATCTAAAGTTGCCCTAAGACTTTCCAGTCCAACTATGTACGGAACAATGCGAGCCTCGTAGCGGAGGCCGCTTCCCGTGGTCACATCTCGTGCCTGACTACTAGTGGACGTAACGGTGGCGCTTGGGAAATCACTGCTTCCGGt       <  1:369711/301‑1 (MQ=255)`  
                                                                                                                                                                                                                                                                                                       `gTATGGAAGGATTGATGACATTAGTAGTACGTACATCATTTCGTGAGGCCATTAAGGACATGCACGAAGAGTATGCAGATAAGGACTCTTTCAAACAATCTCCTGCAACAGTACGGGAGGTGTTCTGATGTCTGACTACCTGAAAGTGCTGCAAGCAATCAAAAGTTGCCCTAAGACTTTCCAGTCCAACTATGTACTGAACAATGCGAGCCTCGTAGCGGAGGCCGCTTCCCGTGGTCACATCTCGTGCCTGACTAATAGTGGACGTCACGGTGGAGCTTGGGAAATCACTGCTTCCGGt       >  1:257084/1‑301 (MQ=255)`  
                                                                                                                                                                                                                                                                                                         `aTGGAAGGATTGATGACATTCGTAGTACGTACATCATTTCGTGAGGCCATTAAGGACATGCACGAAGAGTATGCAGATAAGGACTCTTTCAAACAATCTCCTGCAACAGTACGGGAGGTGTTCTGATGTCTGACTACCCGAAAGTGCTGCAAGCAATCAAAAGTTGCCCTAAGACTTTCCAGTCCAACTATGTACGGAACAATGCGAGCCTCGTAGCGGAGGCCGCTTCCCGTGGTCACATCTCGTGCCTGACTACTAGTGGACGTAACGGTGGCGCTTGGGAAATCACTGCTTCCGGtct     >  1:344820/1‑299 (MQ=255)`  
                                                                                                                                                                                                                                                                                                         `aTGGAAGGATTGATGACATTCGTAGTACGTACATCATTTCATGAGGCCGTTAAGGACATGCACGAAGAGTATGCAGATAAGGACTCTTTCCAACAATCTCCTGCAACAGTACGGGAGGTGTTCTGCTGTCTGACTACCTGAAAGTGCAGCAAGCAATCAAAAGTTGCCCTAAGACTTTCCAGTCCAACTATGTACGTAACAATGCGAGCCTCGTAGCGGAGGCCGCTTCCCGTGGTCACATCTCGTGCCTGACTACTAGTGGACGTAACGGTGGCGCTTGGGAAATCACTGCTTCCGGTAc     <  1:306900/301‑1 (MQ=255)`  

                                                                                                                                                                                                                                                                                                            `|`                                                                                                                                                                                                                                                                                                              
`TGACATTCTTGCGGTTGTGGTTGACGAAGATGTGCTTATTTTCACATGGGACGAAGTAAAAGGTAAGACATCCATGTGTGTCGGCAAGAGAAACAAAGGCATAAAACTATAGGAGAAATTATTATGGCTATGACAAAGAAATTTAAAGTGTCCTTCGACGTTACCGCAAAGATGTCGTCTGACGTTCAGGCAATCTTAGAGAAAGATATGCTGCATCTATGTAAGCAGGTCGGCTCAGGTGCGATTGTCCCCAATGGTAAACAGAAGGAAATGATTGTCCAGTTCCTGACACACGGTATGGAAGGATTGATGACATTCGTAGTACGTACATCATTTCGTGAGGCCATTAAGGACATGCACGAAGAGTATGCAGATAAGGACTCTTTCAAACAATCTCCTGCAACAGTACGGGAGGTGTTCTGATGTCTGACTACCTGAAAGTGCTGCAAGCAATCAAAAGTTGCCCTAAGACTTTCCAGTCCAACTATGTACGGAACAATGCGAGCCTCGTAGCGGAGGCCGCTTCCCGTGGTCACATCTCGTGCCTGACTACTAGTGGACGTAACGGTGGCGCTTGGGAAATCACTGCTTCCGGTACTCG  >  NC_001604.1/16728‑17328`

  

Alignment Legend

Aligned base mismatch/match (shaded by quality score): `ATCG/ATCG` `< 3 ≤` `ATCG/ATCG` `< 10 ≤` `ATCG/ATCG` `< 20 ≤` `ATCG/ATCG` `< 34 ≤` `ATCG/ATCG` `< 38 ≤` `ATCG/ATCG`

Unaligned base: `atcg`    Masked matching base: `atcg`    Alignment gap: `‑`    Deleted base: `‑`

Reads not counted as support for junction

read\_name Not counted due to insufficient overlap past the breakpoint.

read\_name Not counted due to not crossing MOB target site duplication.
